# Prevalence of occult adrenal insufficiency and the prognostic value of a short corticotropin stimulation test in patients with septic shock

**DOI:** 10.4103/0972-5229.56054

**Published:** 2009

**Authors:** Muzaffar Maqbool, Zafar Amin Shah, Fayaz Ahmad Wani, Abdul Wahid, Shaheena Parveen, Arjumand Nazir

**Affiliations:** **From:**1 Department of Internal Medicine, Sheri-Kashmir Institute of Medical Sciences, Srinagar 190 001, J&K, India; 2 Department of Immunology Molecular Medicine, Sheri-Kashmir Institute of Medical Sciences, Srinagar 190 001, J&K, India; 3 Department of Anaesthesia, Sheri-Kashmir Institute of Medical Sciences, Srinagar 190 001, J&K, India

**Keywords:** Adrenal insufficiency, cortisol, mortality, septic shock

## Abstract

**Background:**

Corticosteroid insufficiency in acute illness can be difficult to discern clinically. Occult adrenal insufficiency (i.e., Δmax ≤9 μg/dL) after corticotropin may be associated with a high mortality rate.

**Objective:**

To assess the prevalence of occult adrenal insufficiency and the prognostic value of short corticotropin stimulation test in patients with septic shock.

**Materials and Methods:**

A total of 30 consecutive patients admitted in the adult intensive care unit of the Sheri Kashmir Institute of Medical Sciences who met the clinical criteria for septic shock were prospectively enrolled in the study. A low dose (1 μg) short corticotropin stimulation test was performed; blood samples were taken before the injection (T0) and 30 (T30) and 60 (T60) minutes afterward.

**Results:**

The prevalence of occult adrenal insufficiency was 57%. The 28-day mortality rate was 60% and the median time to death was 12 days. The following seven variables remained independently associated with death: organ system failure scores, simplified acute physiology score II score, mean arterial pressure, low platelet count, PaO_2_:FIO_2_, random baseline cortisol (T0) >34 μg/dL, and maximum variation after test (Δmax) of ≤9 μg/dL. Three different mortality patterns were observed: (I) low (T0 ≤34 μg/dL and Δmax >9 μg/dL; a 28-day mortality rate of 33%),(II) intermediate (T0 >34 μg/dL and Δmax >9 μg/dL or T0 ≤34 μg/dL and Δmax ≤9 μg/dL; a 28-day mortality rate of 71%), and (III) high (T0 >34 μg/dL and Δmax ≤9 μg/dL; a 28-day mortality rate of 82%).

**Conclusion:**

A short corticotropin test using low-dose corticotropin (1 μg) has a good prognostic value. High basal cortisol and a low increase in cortisol on corticotropin stimulation test are predictors of a poor outcome in patients with septic shock.

## Introduction

Septic shock remains the most common cause of death in non coronary intensive care units (ICUs).[1] To tackle this problem, numerous anti-inflammatory therapies have been tested during the past decade, but all of them have been unable to improve survival in patients with severe sepsis.[2] Thus, there is an urgent need to better characterize septic patients with the worst outcome. Several clinical prognostic factors have already been identified (i.e., preexisting underlying disease, presence of organ dysfunction, and severity of illness scores).[3] Moreover, the hormonal profile has been suggested to be a valid predictor of outcome in critically ill patients.[4] However, a pathophysiologic derangement that could help identify a group of patients who might benefit from a particular treatment has not yet been characterized.

Corticosteroid insufficiency in acute illness can be difficult to discern clinically. When the basic features of Addisonian crisis are present, the diagnosis may be obvious, but features such as fever, anorexia, nausea, vomiting, diarrhea, abdominal pain, delirium, and hypotension are common in patients with acute severe illness.[5] In the majority of cases, it remains extremely difficult to recognize adrenal insufficiency in a patient in the intensive care unit. However, important diagnostic clues are hemodynamic instability despite adequate fluid resuscitation (most often associated with a hyperdynamic circulation and decreased systemic vascular resistance) and ongoing evidence of inflammation without an obvious source that does not respond to empirical treatment.[6,7] Limitations of a physical examination suggest that the threshold for investigation should be low, especially in patients with septic shock.

The integrity of the hypothalamic-pituitary-adrenal (HPA) axis is a major determinant of the host's response to stress.[8,9] During sepsis, the activation of the HPA axis is highlighted by increased corticotropin release from the pituitary gland,[10] enhanced adrenal secretory activity,[11,12] and high plasma cortisol levels.[13–16] However, whether endogenous glucocorticoid levels are adequate or constitute an independent predictor of death remains controversial. [13–18] For instance, several studies showed that the higher the plasma cortisol concentrations, the worse the patient's outcome.[4,7,10,19–21] In contrast, other studies reported lower cortisol levels in non survivors compared with survivors.[22–24] For this reason, in severe sepsis, the evaluation of the appropriateness of the activation of the HPA axis requires dynamic testing. In this respect, the most commonly used test is the short corticotropin stimulation test, normal adrenal function being defined by a plasma cortisol level (before or at 30 or 60 minutes after the injection of corticotropin) above 20 μg/dL.[25] However, basal plasma cortisol levels are commonly greater than 20 μg/dL in severe sepsis and the use of the absolute increase in plasma cortisol levels after the intravenous injection of corticotropin may be more useful to evaluate adrenal function.[15,16] Indeed, occult adrenal insufficiency (i.e., an absolute increment of cortisol concentrations ≤ 9 μg/dL) after corticotropin may be associated with impaired pressor responsiveness to norepinephrine[26] and a high mortality rate.[27,28] Such results must be confirmed since other investigators have not found any relationship between cortisol response to corticotropin and survival from sepsis.[29]

So we undertook a prospective study to assess the factors associated with mortality, taking special interest in cortisol levels and cortisol response to corticotropin in patients with septic shock.

### Low-dose ACTH test for the diagnosis of HPA failure

Due to the decreased sensitivity of the high-dose adrenocorticotropic hormone (HD-ACTH) test for diagnosis of adrenal insufficiency, many investigators evaluated the use of stress levels of ACTH (i.e., 1 to 2 μg) for the diagnosis of adrenal insufficiency. A number of studies have demonstrated that a 1 μg dose (low-dose corticotropin [LD-ACTH] stimulation test) of corticotropin is more sensitive and specific for diagnosing primary and secondary adrenal insufficiency than the 250 μg dose of corticotropin (HD-ACTH).[30–34]

## Materials and Methods

### Study Population

A prospective inception cohort study was conducted in 2004 and 2005 in the adult intensive care unit of Sheri Kashmir Institute of Medical Sciences Soura, Srinagar, a tertiary care hospital where most of the modern facilities are available.

Thirty consecutive patients admitted in the adult intensive care unit were prospectively enrolled in the study if they met the following criteria:

1. Informed consent from the next of the kin
2. Presence of septic shock as evidenced by
  - A systemic inflammatory response as defined by two or more of the following: temperature higher than 38.5°C or lower than 35.0°C, heart rate of more than 90/min, respiratory rate of more than 20/min or a need for mechanical ventilation, white blood cell count of more than 12.0 × 10^9^/L or less than 4.0 × 10^9^/L or containing more than 10% immature forms
  - Evidence of a nidus of infection
  - Systolic blood pressure of less than 90 mm Hg (for at least one hour but less than 24 hours) despite adequate fluid replacement and perfusion of 5 μg/kg/min or more of dopamine or dobutamine and the presence of at least two signs of perfusion abnormalities (i.e, acidosis, oliguria, or an abrupt alteration in the mental status)

Patients were excluded from the study if they met any of the following criteria:

1. Had known previous conditions that may have disrupted the HPA axis
2. Were less than 18 years old
3. Had received immunosuppressive therapy
4. Had haematological diseases
5. Were pregnant

The protocol was approved by our institutional ethical committee and informed consent was obtained from the patient's next of kin.

### Clinical Evaluation

At the onset of septic shock, the following variables were recorded:

1. General characteristics including age, gender, date of ICU admission, medical or surgical admission (scheduled or unscheduled)
2. Severity of illness as assessed by the number of organ system failures (OSF score), Simplified Acute Physiology Score II (SAPS II), and vital signs (temperature, mean arterial pressure, heart rate, urinary output.
3. Interventions (at physician's discretion) including volume of fluid infusion per 24 hours, antibiotics, type and titration of vasopressors, surgical procedures, and the need for mechanical ventilation

### Laboratory Variables

At the onset of septic shock, blood cultures and cultures of specimen drawn from the site of infection, hematologic and chemistry data, and blood gas determinations were done systematically. A short corticotropin stimulation test was performed with 1 μg of tetracosactrin (Synacthéne) given intravenously. Blood samples were taken immediately before the test (T0) and 30 (T30) and 60 (T60) minutes afterward. After centrifugation, samples were stored at -20°C until processed. Samples were thawed at room temperature 2-3 hours before processing. Serum cortisol was measured using the Gammacoat (1251) Cortisol Radioimmunoassay Kit (Diasorin Stillwater, Minnesota 55082, USA) based on the competitive binding principle of the radioimmunoassay. The cortisol response to the corticotropin stimulation test (Δmax) was defined as the difference between T0 and the highest of the T30 and T60 concentrations.

### Follow-up

All patients were evaluated for 28 days after inclusion in the study. The evaluation of the following variables was performed daily in each patient during shock: vital signs, arterial blood gases, plasma electrolytes, glucose levels, serum creatinine and liver function tests, and interventions as previously defined.

### Statistical Analysis

The statistical analysis of the data was done using student's t-test for the difference of means and a chi-square test and Fishers Exact test were used for nominal data. These tests were referred for *P*-values for their statistical significance. The *P*-values (*P*<0.05) were considered statistically significant. The analysis was performed using comprehensive statistical software, Version 10.0 (Chicago, USA) for Windows^®^

## Results

### Patient Characteristics

A total of 30 patients were admitted during the study period in the intensive care unit of Sheri Kashmir Institute of Medical Sciences Soura, Srinagar- a tertiary care hospital where most of the modern facilities are available. Of the 30 patients, 18 (60%; 95% CI, 53–67%) died within the 28-day period following the onset of septic shock. As noted in Table 1, the mean age in years of the survivors and non survivors was 47.25 years old (SD=16.62) and 58.72 years old (SD=19.63), respectively. This difference is statistically non significant (*P*= 0.108). Also noted in the same table is that there is a statistically non significant difference (*p*=0.534) in the distribution of gender with respect to survival in patients with septic shock.

**Table 1:** Age and gender distribution of patients with respect to survival in septic shock (p-value is for a comparison between survivors and non survivors)

	Mean age (years)	Male (n=19)	Female (n=11)	Total (n=30)
Survivors	47.25	8 (42.11%)	4 (36.36%)	12
Non survivors	58.72	11 (57.89%)	7 (63.64%)	18
P-value	0.108	0.534	

Tables 2 and 3 show the patient characteristics at the onset of septic shock and the results of the analysis between the groups of survivors and non survivors. Severity assessment scores, OSF scores (*P*=0.000), and the SAPS II (*P*= 0.000) were significantly associated with mortality.

**Table 2:** Clinical and laboratory parameters at onset of septic shock

Variable	Survivors (n= 12)	Non survivors (n= 18)	P-value
Pulse beats/min	112.50(18.51)	112.56(19.96)	0.994
Temperature(0C)	38.57(1.05)	38.13(0.96)	0.245
Respiratory Rate (breaths/min)	31.50(6.33)	34.22(7.25)	0.299
MAP (mmHg)	71.42(4.74)	58.61(9.62)	0.000
Hb (g/dl)	11.73(1.96)	11.42(2.43)	0.717
TLC (×109/L)	15.73(3.16)	15.48(3.60)	0.846
Platelets (×109/L)	184(66.49)	113.17(67.24)	0.008
Creatinine (mg/dl)	1.82(0.75)	3.58(2.09)	0.010
Bilirubin (mg/dl)	1.62(1.19)	2.49(2.31)	0.240
paO2: FiO2 (mmHg)	222(114)	157(102)	0.000

**Table 3:** Distribution of Organ System Failure Assessment (OSFA) Score and SAPS II Score with respect to survival in patients with septic shock

OSFA	Score	Total (n=30)	Survivors (n=12)	Non survivors (n=18)	*P* value
<2	0	1	1(100)	0(0)	
	1	9	8(89)	1(11)	0.000
≥2	2	5	2(40)	3(60)	
	3	8	1(12.5)	7(87.5)	
	4	5	0(0)	5(100)	
	5	2	0(0)	2(100)	
SAPS II Score		49.17 (13.29)	39.42 (6.22)	55.67 (12.82)	0.000

Among clinical and biological factors, mean arterial pressure, platelet count, serum creatinine, and the ratio of the PaO_2_ to the fraction of inspired oxygen (FIO_2_) were significantly different between the survivors and non survivors. Compared with the survivors, the non survivors had significantly higher basal plasma cortisol levels (T0) and lower cortisol response to corticotropin (Δmax).

The number of patients who had documented infection and strains diagnosed at the onset of septic shock are shown in Table 4. Gram-positive microorganisms were more common among non survivors and gram-negative microorganisms were more common among survivors, but the difference was statistically not significant (*P*= 0.200). In 5 patients, both gram-positive and gram-negative organisms were isolated.

**Table 4:** Types of microorganisms isolated from patients at the onset of septic shock

Microorganism	Total N=35	Survivors N=17	Non-survivors N=18	*P* value
Gram-Positive	18(51%)	7(41%)	11(61%)	0.200
Gram-Negative	17(49%)	10(59%)	7(39%)	

The median time to death was 12 days for all patients. The prevalence of occult adrenal insufficiency was 57% (95% CI, 50–64%).

Among the variables, as shown in Tables 3–6, the following seven remained independently associated with death: OSF scores greater than two (*P*=0.000), SAPS II greater than 55 (*P*=0.000), mean arterial pressure of 60 mm Hg or less (*P*=0.000), low platelet count (*P*=0.008), PaO_2_:FIO_2_ < 160 mmHg (*P*=0.001), random baseline cortisol (T0) greater than 34 μg/dL (*P*=0.014), and maximum variation after test (Δmax) of ≤9 μg/dL (*P* =0.002).

### Cortisol Levels and Cortisol Response to Corticotropin

The random baseline cortisol levels (T0) and response to corticotropin stimulation test (Δmax) for patients with septic shock (survivors and non survivors) are shown in Table 5. The mean serum baseline cortisol concentrations in survivors and non survivors was 28 μg/dL (±SD 5.46) and 38.66 μg/dL (±SD 13.33), respectively. This difference is statistically significant (*P*=0.014). Similarly, non survivors showed a significantly inadequate response to the low dose corticotropin stimulation test as compared with survivors (Δmax = 7.3±3.66 μg/dL in non survivors *vs.* 13±5.28 μg/dL in survivors; *p*=0.002), thereby showing that patients in septic shock who had higher basal serum cortisol levels (>34 μg/dL) and an inadequate response to the corticotropin stimulation test (Δm ≤ 9 μg/dL) had less chance of survival.

**Table 5:** Random cortisol levels and response to short corticotropin stimulation test in patients with septic shock

Cortisol (μg/dL)	Total (n=30)	Survivors (n=12)	Non survivors (n=18)	*P* value
Level before test (T0)	34.40 (11.99)	28 (5.46)	38.66 (13.33)	0.014
Maximum variation after test (max)	9.61 (5.13)	13 (5.28)	7.35 (3.66)	0.002

Therefore, the following combinations of T0 and Δmax were studied:

1. T0 of 34 μg/dL or less and Δmax of more than 9 μg/dL
2. T0 of 34μg/dL or less and Δmax of 9 μg/dL or less or a T0 greater than 34 μg/dL and Δmax more than 9 μg/dL
3. T0 greater than 34 μg/dL and Δmax of 9 μg/dL or less

The information provided by T0 and Δmax together was significantly associated with death rates and distribution of survival times.

Death occurred more rapidly for patients with T0 greater than 34 μg/dL and Δmax of 9 μg/dL or less. Three different mortality patterns appear in Table 6: (I) low (T0 ≤34 μg/dL and Δmax >9 μg/dL; 28-day mortality rate of 33%), (II) intermediate (T0 >34 μg/dL and Δmax >9μg/dL or T0 ≤ 34 μg/dL and Δmax ≤9 μg/dL); 28-day mortality rate of 71%), and (III) high (T0 >34 μg/dL and Δmax ≤9 μg/dL; 28-day mortality rate of 82%).

**Table 6:** Prognostic value of cortisol levels and cortisol response to corticotropin

Group	Basal cortisol level (T0)	Increase in cortisol levels (Δmax)	28-day mortality rate
I	≤ 34 *μ*g/dL	> 9 *μ*g/dL	33%
II	> 34 *μ*g/dL	> 9 *μ*g/dL	71%
	≤ 34 *μ*g/dL	≤ 9 *μ*g/dL	
III	> 34 *μ*g/dL	≤ 9 *μ*g/dL	82%

## Discussion

In this study, 30 consecutive patients admitted in the adult intensive care unit with well-defined diagnosis of septic shock, complete clinical and physiological data, and a complete follow-up were included. The study was mainly designed to assess, at the early course of septic shock, the prognostic value of occult adrenal insufficiency.

In our study, the 28-day mortality rate from septic shock was 60% (95% CI, 53–67%). This result is consistent with the 56% rate of ICU mortality at 28 days reported in literature.[3] The prevalence of occult adrenal insufficiency, in our patients with septic shock, was 57% (95% CI, 50–64%). This is consistent with the results of Annane, *et al*.[35] However, the results are lower than 81.6% reported by Chacko, *et al*.[36] Earlier literature quotes a wide range in the incidence of hypocortisolemia in the critically ill; this may be attributable to the different types of illnesses encountered from center to center.[36]

Several factors have been suspected to be associated with mortality in cases of severe sepsis and septic shock.[3,37–42] The main prognostic factors reported to date are age, severity of the patient's underlying disease, number of organ system dysfunctions, severity of illness scores, hypothermia, neutropenia, thrombocytopenia, lactic acidosis, multisource of infection, positive blood culture, type of infecting organism, blood concentrations of endotoxin, and cytokines. In our study, we found seven factors remained significantly associated with death: OSF scores greater than two (*P*=0.000), SAPS II greater than 55 (*P*=0.000), mean arterial pressure of 60 mm Hg or less (*P*=0.000), low platelet count (*P*=0.008), PaO_2_:FIO_2_ < 160 mmHg (*P* =0.001), random baseline cortisol (T0) greater than 34 μg/dL (*P* =0.014), and maximum variation after test (Δmax) of ≤ 9 μg/dL (*P* =0.002).

In the present study, the age and gender distribution of patients with septic shock was comparable between survivors and non survivors (*P*=0.108 and 0.534, respectively). The mean age of survivors and non survivors was 47.25 years old (±SD 16.62) and 58.72 years old (±SD 19.63), respectively. Survival rates in males and females were 42.1% and 36.36%, respectively. These findings are consistent with the findings of Annane, *et al.*[43] No significant difference (*P*=0.350) was found in the type of admission – whether medical or surgical in patients with septic shock.

Vital signs such as pulse, temperature, and respiratory rate were comparable in survivors and non survivors (*P*=0.994, 0.245 and 0.299, respectively). The mean ± SD mean arterial pressure (MAP) in mmHg was significantly different (*P*=0.000) in survivors and non survivors. The severity assessment scores, OSF scores, and SAPS II scores were significantly different between survivors and non survivors (*P*=0.000). An OSF score >2 was significantly associated with mortality. The findings are consistent with those reported in other studies.[43] Laboratory parameters like Hb, TLC, and bilirubin were comparable between survivors and non survivors (*P*=0.717, 0.846 and 0.245, respectively). However, a significant difference was observed in the distribution of platelets (0.008), creatinine (0.010), and the ratio of PaO_2_ to FiO_2_ (0.000) between survivors and non survivors.

This study showed that the mean serum baseline cortisol concentration in survivors and non survivors was 28 (±SD 5.46) and 38.66 (± SD 13.33), respectively. This difference is statistically significant (*P* =0.014). Chacko, *et al.* have found baseline serum cortisol levels of 23.24, which is lower than our study.[36] However, they have not compared the data between survivors and non survivors. The higher baseline serum cortisol found in our study could be because our patients could have been more sick and so more stressed, as plasma cortisol levels seem to reflect the severity of illness.[12] Similarly, non survivors showed significantly inadequate response to the low-dose corticotrophin stimulation test as compared with the survivors (Δmax= 7.35 ± 3.66 in non survivors *vs.* 13 ± 5.28 μg/dL in survivors; *P* =0.002), thereby showing that patients in septic shock who had higher baseline serum cortisol levels (>34 μg/dL) and inadequate response to the corticotrophin stimulation test (Δmax ≤ 9 μg/dL) had fewer chances of survival. These findings are consistent with previous studies.[29,44,45]

Thus, at the onset of septic shock, basal plasma cortisol values and cortisol response to corticotropin appear to be independent predictors of 28-day mortality, which allows us to substantiate the prognostic classification of 3 groups given by Annane, *et al*.[43] This classification requires only a short corticotropin test and has a good prognostic value. It should therefore be helpful in identifying a group of patients at high-risk for death and who may be benefited by treatment with corticosteroids. The role of corticosteroids in patients of septic shock with relative adrenal insufficiency has also been highlighted in an editorial by Mani.[46]

## Conclusion

As per our study, a short corticotropin test using low-dose corticotropin (1 μg) has a good prognostic value and can be helpful in identifying patients with septic shock at high-risk for death. High basal cortisol levels and a low increase in cortisol on a corticotropin stimulation test are predictors of a poor outcome in patients with septic shock. The prevalence of occult adrenal insufficiency, using a low dose (1 μg) corticotropin stimulation test is 57% in patients with septic shock. The 28-day mortality rate for patients with septic shock is 60%.
